# Radiofrequency Irradiation Modulates TRPV1-Related Burning Sensation in Rosacea

**DOI:** 10.3390/molecules26051424

**Published:** 2021-03-06

**Authors:** Seyeon Oh, Myeongjoo Son, Joonhong Park, Donghwan Kang, Kyunghee Byun

**Affiliations:** 1 Functional Cellular Networks Laboratory, Lee Gil Ya Cancer and Diabetes Institute, Gachon University College of Medicine, Incheon 21999, Korea; seyeon8965@gmail.com (S.O.); mjson@gachon.ac.kr (M.S.); 2 Department of Anatomy & Cell Biology, Gachon University College of Medicine, Incheon 21936, Korea; 3 Maymorning Dermatologic Clinic, Sungnam 13306, Korea; 5maymorning@gmail.com; 4 Jeisys Medical Inc., Seoul 08501, Korea; kang@jeisys.com

**Keywords:** rosacea, burning sensation, microneedling radiofrequency, TRPV1, neuropeptides

## Abstract

Rosacea is a skin inflammatory condition that is accompanied by not only redness and flushing but also unseen symptoms, such as burning, stinging, and itching. TRPV1 expression in UVB-exposed skin can lead to a painful burning sensation. Upregulated TRPV1 expression helps release neuropeptides, including calcitonin gene-related peptide, pituitary adenylate cyclase-activating polypeptide, and vasoactive intestinal peptide, which can activate macrophage and inflammatory molecules. In this study, we found that radiofrequency (RF) irradiation reduced TRPV1 activation and neuropeptide expression in a UVB-exposed in vivo model and UVB- or heat-treated in an in vitro model. RF irradiation attenuated neuropeptide-induced macrophage activation and inflammatory molecule expression. Interestingly, the burning sensation in the skin of UVB-exposed mice and patients with rosacea was significantly decreased by RF irradiation. These results can provide experimental and molecular evidence on the effective use of RF irradiation for the burning sensation in patients with rosacea.

## 1. Introduction

Rosacea is a skin inflammatory condition that is accompanied by not only redness and flushing but also unseen symptoms, such as burning, stinging, and itching [1]. These symptoms, especially a facial burning sensation, make patients with rosacea feel more pain. Transient receptor potential vanilloid subfamily (TRPV) receptors, which are known to be closely related to burning sensations, comprise six subfamilies (TRPV1–TRPV6); four subfamilies (TRPV1 to TPRV4) are nonselective cation channels, whereas two subfamilies (TRPV5 and TRPV6) are calcium-selective channels [2,3]. Among the TRPV subfamilies, TRPV1 participates in cutaneous neurogenic inflammation and pain as a nociceptive cationic channel, mainly for Ca^2+^, and can be activated by various rosacea trigger factors, including osmotic or pH changes, high temperature exposure (above 43 °C), and ultraviolet B (UVB) light exposure [3,4,5]. Some TRPV1 gene knockout studies have shown reductions in inflammatory pain [6,7], and in heat pain [8]. In addition to the perception of pain, other skin-relevant neuronal functions, such as itch-related scratching behavior, are affected by TRPV1 [9]. Interestingly, the distribution and expression of the TRPV subfamilies vary among the various rosacea subtypes. TRPV1 expression level on skin biopsy was reported to be higher in patients with erythematotelangiectatic and phymatous rosacea than in healthy controls [10].

TRPV1 is a polymodal receptor with sensitization and endogenous regulatory pathways that act through phosphorylation sites for kinases, such as protein kinase C (PKC), protein kinase A, and Ca^2+^/calmodulin-dependent kinase II (CAMKII) [11]. TRPV1 is profusely expressed in the sensory nerve endings of the skin, leading to the release of neuropeptides, including calcitonin gene-related peptide (CGRP), pituitary adenylate cyclase-activating polypeptide (PACAP), and vasoactive intestinal peptide (VIP) [5,12,13,14]. CGRP was reported to be a key regulator of cutaneous immunity; is associated with various inflammatory diseases, including diabetes, sepsis, and Crohn’s Disease [15]; and influences the expression of cAMP suppressor genes and NF-κB in immune cells [16]. Similar to CGRP, intradermal injections of PACAP38 and VIP were shown to increase pain intensity and skin blood flow and led to larger flares and wheals in a human acute cutaneous pain model [17].

Radiofrequency (RF) irradiation had been shown by several studies to be clinically effective for rosacea [18,19,20], but its molecular effects have been reported by only few experimental studies. Recently, we reported that RF irradiation reduced keratinocyte proliferation in the epidermal layer and the expressions of proinflammatory cytokines and angiogenesis-related factors in vitro and in vivo. RF irradiation attenuated VEGF-induced angiogenesis-associated processes, such as tube formation, cell migration, and endothelial cell proliferation. Notably, the blood vessel densities in the skins of UVB-treated mice and patients with rosacea were significantly decreased by RF irradiation. However, we did not validate the changes in burning sensation in patients with rosacea. In this present study, we investigated whether RF irradiation can mediate the expressions of important TRPV1- and TRPV1-mediated neuropeptides, including CGRP, PACAP, and VIP, which are active in the skin of mice with UVB-exposure rosacea (in vivo) and UVB- or heat-treated sensory neuronal cells (in vitro). In addition, we found that RF irradiation attenuated facial temperature and burning sensations in patients with rosacea.

## 2. Results

### 2.1. The Attenuating Effects of RF Irradiation on TRPV1 and Its Related PKC-Dependent Pathway in UVB-Exposed Mice Skin and UVB- or Heat-Treated Sensory Neuronal Cells

**Figure 1:**
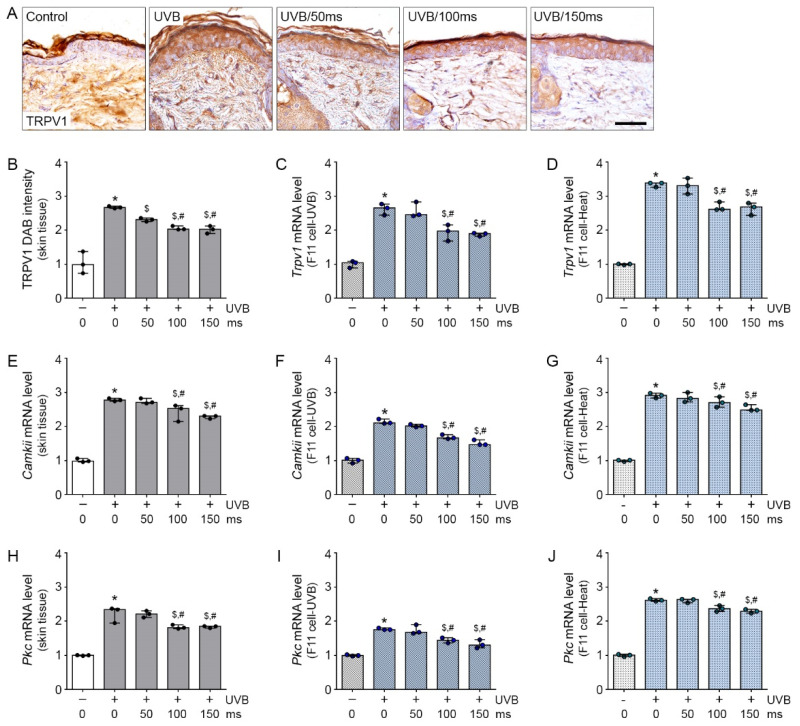
Inhibitory effects of RF on TRPV1 and PKC-dependent pathway in UVB-exposed mice skin and UVB- or heat-treated sensory neuronal cells. (**A**,**B**) TRPV1 expression level was analyzed by immunohistochemistry (3 mice per group). Scale bar = 100 µm; (**B**–**J**) Expression of the TRPV1 related PKC-dependent pathway molecule, *CAMKII* and *PKC* mRNA were confirmed by qRT-PCR in the skin tissue (**B**,**E**,**H**), UVB exposure F11 cell model (**C**,**F**,**I**) and heat exposure F11 cell model (**D**,**G,J**) and were normalized to that of *Actb* and expressed relative to the control group. * *p* < 0.05 vs. Control; ^$^ *p* < 0.05 vs. UVB or Heat; ^#^ *p* *<* 0.05 vs. RF 50 ms.

UVB exposure was shown to upregulate TRPV1 expression in skin tissue [13]. To validate the effective RF irradiation that will reduce TRPV1 expression in vivo, we applied RF irradiation on the skin of UVB-exposed mice. UVB radiation significantly increased TRPV1 protein expression in UVB-irradiated mice skin (UVB group) than in the non UVB-irradiated mice skin (control group). TRPV1 expression was significantly lower in RF-irradiated UVB-exposed mice skin (UVB/RF group) than in the UVB group; this effect was not significantly different among the RF irradiation durations 50 (UVB/50), 100 (UVB/100), and 150 (UVB/150) millisecond (ms) (Figure 1A,B). In addition, we made an in vitro model of rosacea to mimic and environment of increased Trpv1 mRNA caused by UVB or heat exposure to F11 cells, which are sensory neuronal cells. 

For the F11 cells, UVB treatment significantly increased Trpv1 mRNA expression in the UVB group compared with the control group. Trpv1 mRNA expression was significantly lower in the UVB/RF group than in the UVB group; this effect was not significantly different between the RF irradiation durations UVB/100 and UVB/150 ms (Figure 1C). Similar to our experiments on the UVB-exposed in vivo and in vitro models, the Trpv1 mRNA expression was validated in the heat-treated F11 cell in the in vitro model. In the heat-treated F11 cell in vitro model, the increased level of Trpv1 by 45 °C heat treatment was significantly decreased by RF irradiation; this effect was most prominent at 100 and 150 ms of RF irradiation (Figure 1D).

UVB or heat treatment activates the neuropeptide expression of sensory neurons through the TRPV1-PKC pathway. To determine the effects and mechanism of RF irradiation on the regulation of the TRPV1-PKC pathway in a UVB- or heat-treated condition, we examined the two calcium binding proteins CAMKII and PKC. Activated TRPV1 increased CAMKII, which is an important molecule in the TRPV1-PKC pathway [21]. The Camkii mRNA expression level in mice skin was significantly higher in the UVB group than in the control group; this effect was most prominent at 100 and 150 ms of RF irradiation and was significantly decreased by RF irradiation (Figure 1E).

In the F11 cells, the expression of Camkii mRNA was significantly higher in the UVB group than in the control group; this effect was most prominent and significantly decreased by 100 and 150 ms of RF irradiation (Figure 1F). The expression of Camkii mRNA in F11 cells was significantly increased by heat treatment, compared with that without heat treatment; this effect was significantly decreased by RF irradiation, but there was no significant difference between the 100 and 150 ms of RF irradiation (Figure 1G).

UVB radiation significantly increased the Pkc mRNA expression in the mice skin of the UVB group compared to that of the control group. Pkc mRNA expression was significantly lower in the UVB/RF group than in the UVB group; the decreasing effect was not significantly different between 100 and 150 ms of RF irradiation (Figure 1H). In the in vitro studies, UVB treatment significantly increased Pkc mRNA expression in the UVB group compared with the control group. Pkc mRNA expression was significantly lower in the UVB/RF group than in the UVB group; this effect was not significantly different between 100 and 150 ms of RF irradiation (Figure 1I). The increased level of Pkc mRNA by heat treatment was significantly decreased by RF irradiation; this effect was most prominent at 100 and 150 ms of RF irradiation (Figure 1J). Interestingly, RF irradiation for a short time (i.e., 50 ms) had no TRPV1 reduction effect on the UVB exposure in vivo model and UVB- or heat-treated in vitro model. However, among three conditions, prolonged RF irradiation (i.e., 100 and 150 ms) was shown to have TRPV1 reduction effects. Therefore, RF irradiation attenuated the increased expression of TRPV1 in UVB-exposed mice skin and in UVB- or heat- treated F11 cells. In addition, Capsaicin as well-known as a TRPV1 agonist treatment increased Trpv1, Camkii and Pkc mRNA levels in F11 cells, but RF irradiation revealed those expression levels (supplementary methods and materials and Figure S1).

### 2.2. Inhibitory Effects of RF Irradiation on TRPV1-Induced Neuropeptide Expression

**Figure 2:**
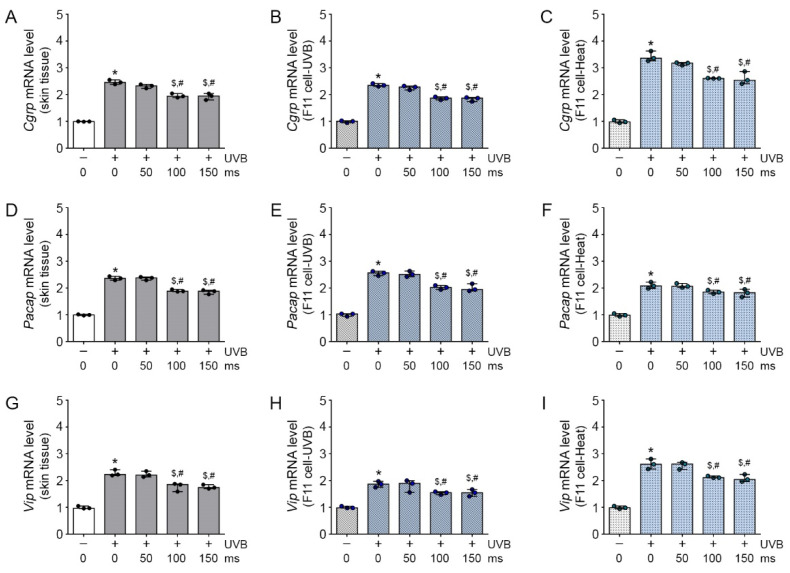
Inhibitory effects of RF on TRPV1-induced neuropeptide expression in UVB-exposed mice skin and UVB- or heat-treated sensory neuronal cells. (**A**–**I**) The TRPV1-induced neuropeptide (*Cgrp, Pacap, and Vip*) expression level was analyzed by qRT-PCR in the skin tissue (**A**,**D**,**G**) (3 mice per group), UVB exposure F11 cell model (**B**,**E**,**H**) and heat exposure F11 cell model (**C**,**F**,**I**) and were normalized to that of *Actb* and expressed relative to the control group. * *p* < 0.05 vs. Control; ^$^ *p* < 0.05 vs. UVB or Heat; ^#^ *p <* 0.05 vs. RF 50 ms.

TRPV1 activation allows the release of various neuropeptides, including CGRP, PACAP, and VIP, which are mainly in the skin [13]. The Cgrp, Pacap, and Vip mRNA expression on UVB-exposed mice skin were significantly increased, compared with those in control mice skin. The increased Cgrp, Pacap, and Vip expressions were significantly decreased by RF irradiation (Figure 2A,D,G), and this effect was not significantly different between 100 and 150 ms of RF irradiation. As expected, the expressions of Cgrp, Pacap, and Vip mRNA in the F11 cells were significantly higher in the UVB group than in the control group and significantly lower in the UVB/RF group than in the UVB group (Figure 2B,E,H). Similar to the results in the UVB-treated F11 cells, the Cgrp, Pacap, and Vip mRNA expressions were significantly higher in the heat group than in the control group and significantly lower in the heat/RF group than in the heat-treated F11 cells (Figure 2C,F,I). In addition, Capsaicin treatment increased Cgrp, Pacap, and Vip mRNA expressions in F11 cells, but RF irradiation revealed those expression levels (Figure S2). Therefore, RF irradiation attenuated the increased mRNA expression of the neuropeptides Cgrp, Pacap, and Vip in UVB-exposed mice skin and UVB-, heat-or capsaicin- treated F11 cells.

### 2.3. Effects of RF Irradiation on Neuropeptide-Induced Inflammation

**Figure 3:**
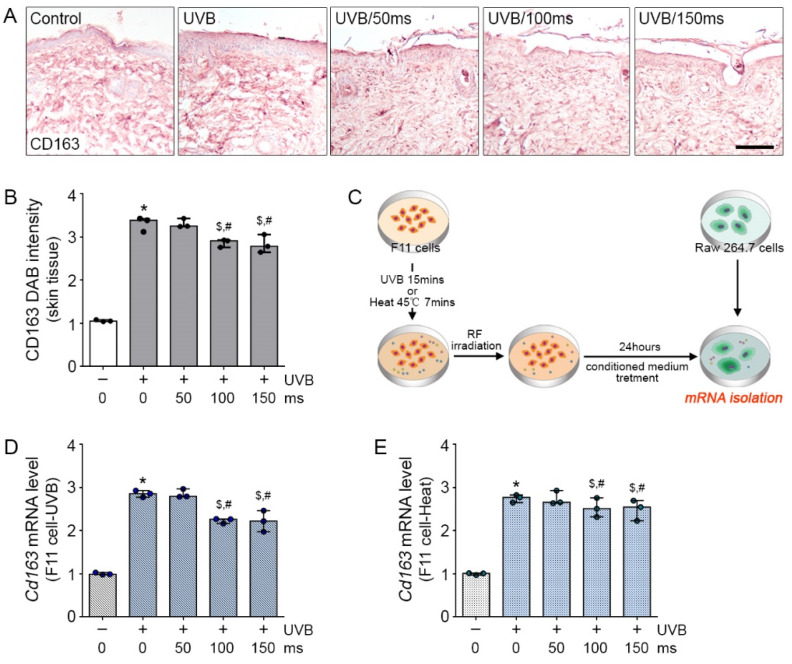
Inhibitory effects of RF on neuropeptide-induced macrophage activation in UVB-exposed mice skin and UVB- or heat-treated sensory neuronal cells. (**A**,**B**) The CD163 expression level was analyzed by immunohistochemistry. Scale bar = 100 µm; (3 mice per group) (**C**) The cell experimental scheme for Raw264.7. affected by F11 cells; (**D**,**E**) mRNA expression of the *Cd163* by qRT-PCR in UVB-CM exposure Raw264.7 cell model (**D**) and heat-CM exposure Raw264.7 cell model (**E**) and were normalized to that of *Actb* and expressed relative to the control group. * *p* < 0.05 vs. Control; ^$^ *p* < 0.05 vs. UVB-CM or Heat-CM; ^#^ *p* < 0.05 vs. RF 50 ms.

Secreted neuropeptides from dermal sensory neurons can induce skin inflammation. To validate whether the various neuropeptides released from TRPV1-activated sensory neuronal cells can cause skin inflammation, we first confirmed macrophage activation in UVB-exposed skin. The expression of CD163, which is a well-known superior dermal macrophage marker [22], can identify a subpopulation of classically activated macrophages in skin diseases, such as psoriasis. The increased CD163 expression by UVB exposure in mice skin was significantly decreased by RF irradiation (Figure 3A,B); this effect was not significantly different between 100 and 150 ms of RF irradiation. In addition, we made indirect in vitro systems using UVB- or heat-treated F11 cells. As shown in Figure 3C, the UVB- or heat-treated F11 cells were shown to secrete some neuropeptides, including CGRP, PACAP, and VIP, which can modulate macrophage activation.

The conditioned medium (CM) from UVB- or heat-treated F11 cells were incubated with macrophage cells (Raw 264.7 cells) for 24 h to validate changes in Cd163 expression. Compared with the CM from non UVB-treated F11 cells incubated with Raw 264.7 cells (CM-treated macrophages or CM group), the CM from UVB-treated F11 cells incubated with Raw 264.7 cells (UVB-CM-incubated Raw 264.7 cells or UVB-CM group) had significantly higher Cd163 expression and the CM from RF with UVB-treated F11 cells incubated with Raw 264.7 cells (RF with UVB-CM-incubated Raw 264.7 cells or UVB-RF/CM group) had significantly lower Cd163 mRNA expression (Figure 3D). As expected, compared with the CM from non-heat-treated F11 cells incubated with Raw 264.7 cells (CM-treated macrophages or CM group), the CM from heat-treated F11 cells incubated with Raw 264.7 cells (heat-CM-incubated Raw 264.7 cells or heat-CM group) had significantly higher Cd163 mRNA expression and the CM from RF with heat-treated F11 cells incubated with Raw 264.7 cells (RF with heat-CM-incubated Raw 264.7 cells or heat-RF/CM group) had significantly lower Cd163 mRNA expression (Figure 3E).

**Figure 4:**
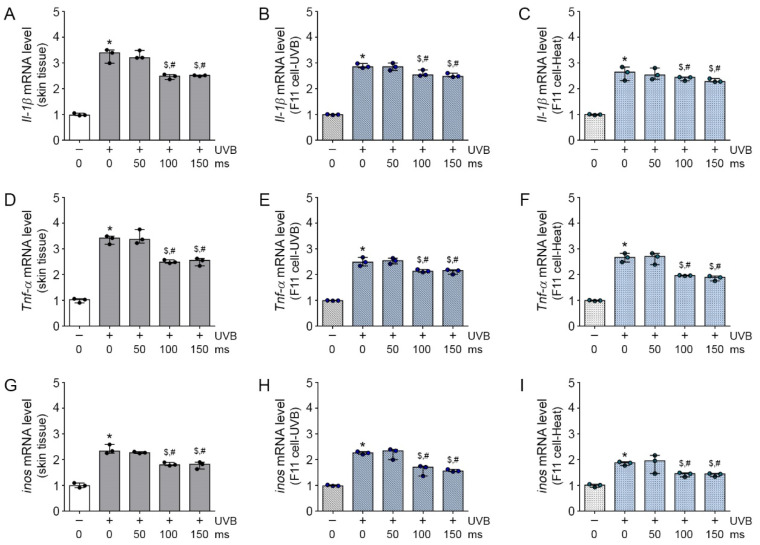
Attenuated effects of RF on neuropeptide-induced inflammation in UVB-exposed mice skin and UVB- or heat-treated sensory neuronal cells. (**A**–**I**) The neuropeptide-induced inflammation cytokine (*Il-1β,* *Tnf-α,* and *inos*) mRNA expression level was analyzed by qRT-PCR in the skin tissue (**A**,**D**,**G**) (3 mice per group), UVB-CM-treated Raw264.7 cell model (**B**,**E**,**H**) and heat-CM treated Raw264.7 cell model (**C**,**F**,**I**) and were normalized to that of *Actb* and expressed relative to the control group. * *p* < 0.05 vs. Control; ^$^ *p* < 0.05 vs. UVB-CM or Heat-CM; ^#^ *p <* 0.05 vs. RF 50 ms.

In addition, we validated the expression of inflammatory molecules, including IL-1β, TNF-α, and iNOS, on CD163 [21]. The increased Il-1β, Tnf-α, and inos mRNA expressions by UVB exposure in mice skin were significantly decreased by RF irradiation; this effect was not significantly different between 100 and 150 ms of RF irradiation (Figure 4A,D,G). The Il-1β, Tnf-α, and inos mRNA expressions were significantly higher in the UVB-CM group and significantly lower in the UVB-RF/CM group than in the CM group (Figure 4B,E,H). As expected, the Il-1β, Tnf-α, and inos mRNA expressions were significantly higher in the heat-CM group and significantly lower in the heat-RF/CM group than in the CM group (Figure 4C,F,I); these effects were most prominent at 100 and 150 ms of RF irradiation. Interestingly, similar to the results on TRPV1 expression, RF irradiation for a short time had no effects on macrophage activation and inflammation modulation, but prolonged RF irradiation decreased macrophage activation and increased inflammation modulation effects. Therefore, RF irradiation attenuated the increase in macrophage activation and inflammation in UVB-exposed mice skin and UVB- or heat-treated F11 cells.

### 2.4. Effects of RF Irradiation on the Burning Sensation in Patients with Rosacea

**Figure 5:**
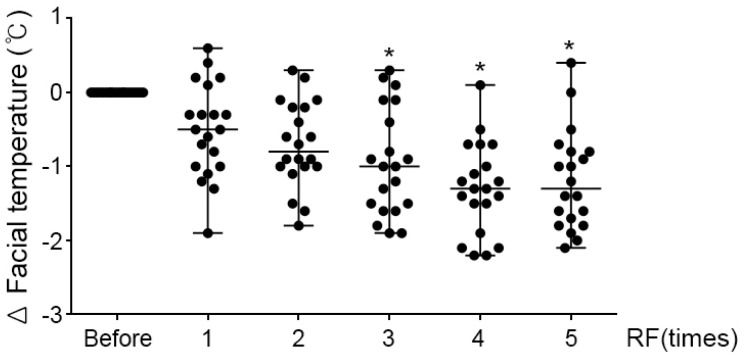
Effects of RF irradiation on the burning sensation in patients with rosacea. The burning sensation in patients with rosacea was measured on facial temperature after RF irradiation 1 to 5 times. The graph showed the results of measurements before the procedure (before) and the amount of temperature change (delta) measured after each RF procedure (1st-5th). * *p* < 0.05 vs. before RF irradiation.

The dermal burning sensation in many patients with rosacea is brought about by various causes, such as skin inflammation and vasodilation. RF irradiation showed clinical effects by suppressing the burning sensation in patients with rosacea. As exemplified in Figure 5, the patient measured the surface temperature of the face before the RF procedure, and the result of measuring the face after the procedure 5 times (5th) compared to the measurement before the RF procedure (before) showed that the overall surface temperature of the face decreased by 1.19 ± 0.67 °C. In summary, we found that RF irradiation significantly attenuated TRPV1-induced neuropeptide expression and dermal inflammation in UVB-treated mice skin and UVB- or heat-treated sensory neuronal cells. Similar to mice skin, the skin of patients with rosacea had a reduced burning sensation after RF irradiation.

## 3. Discussion

Facial redness and burning sensation are the main symptoms in patients with rosacea. In a previous study, we showed that RF irradiation can attenuate VEGF-induced angiogenesis processes, such as endothelial cell proliferation, tube formation, and migration (in vitro) and can modulate increased blood vessel density in UVB-treated mice (in vivo) and patients with rosacea [23]. However, the effect and cellular mechanism of RF irradiation in reducing the burning sensation on the skin have rarely been reported.

The activation of TRPV1 leads to a painful burning sensation. Studies that showed reduction in burning sensation in rosacea with a TRPV1 antagonist are not many. The TRPV1 antagonist PAC-14028 was shown to accelerate skin barrier recovery after tape stripping and in two models of atopic dermatitis [24,25,26]. In a study on hairless mice, another TRPV1 antagonist, iodoresiniferatoxin, was shown to suppress UVB-induced skin thickening and the expressions of MMPs, COX2, and p53 [27]. However, TRPV1 antagonists have no effects on body temperature [28,29]. In addition, some neuropeptides, such as CGRP, PACAP, VIP, and substance P, have properties that are closely related with the pathogenesis of rosacea and are increased in that condition [30]. CGRP is one of the most potent microvascular vasodilators found in human skin; it enhances local inflammation as a consequence of increased blood flow and has the ability to modulate cell activities [31,32]. PACAP can stimulate endothelial cell NO release, which results in indirect vasodilation [33]. Neuropeptides activate mast cells to release histamine, which induces vasodilation, and tryptase, which is a chemotactic agent for fibroblasts and matrix metalloproteinases (MMPs); these contribute to fibrosis in rosacea [30].

In this study, the TRPV1-induced expression of neuropeptides was significantly decreased by prolonged RF irradiation for 100 and 150 ms. Based on these results, we selected 10 W for 100 ms as the golden parameter to effectively control the important factor for regulation of burning sensation (Figure 1 and Figure 2). In our previous in vitro and in vivo studies, we have previously used 15 types of RF irradiation conditions (2 MHz; 8–12 Watts; 50, 100 or 150 ms) and found reduced blood vessel density, even in human patients with rosacea. The RF conditions used in this study were selected based on those previous results [23].

The CD163 expressed in macrophages play a key role in the etiology of rosacea. The number of CD163-positive cells was reported to be higher in the rosacea subtypes erythematotelangiectatic, papulopustular, and phymatous rosacea than in healthy skin, and CD163 expression was highest in papulopustular rosacea [34]. As expected, the CD163-positive macrophages produce inflammatory molecules IL-23/p19 and IL-12/23p40, tumor necrosis factor (TNF), and inducible nitric oxide synthase (iNOS), which can exacerbate skin diseases [22]. In this study, RF irradiation reduced the expressions of CD163 and the related inflammatory molecules in UVB-exposed mice skin and UVB- or heat-CM-treated Raw 264.7 cells (Figure 3 and Figure 4).

As a limitation of this study, expression levels of the cell signaling pathway related molecules are measured from mRNA and the results can be overinterpreted in vitro and in vivo.

**Figure 6:**
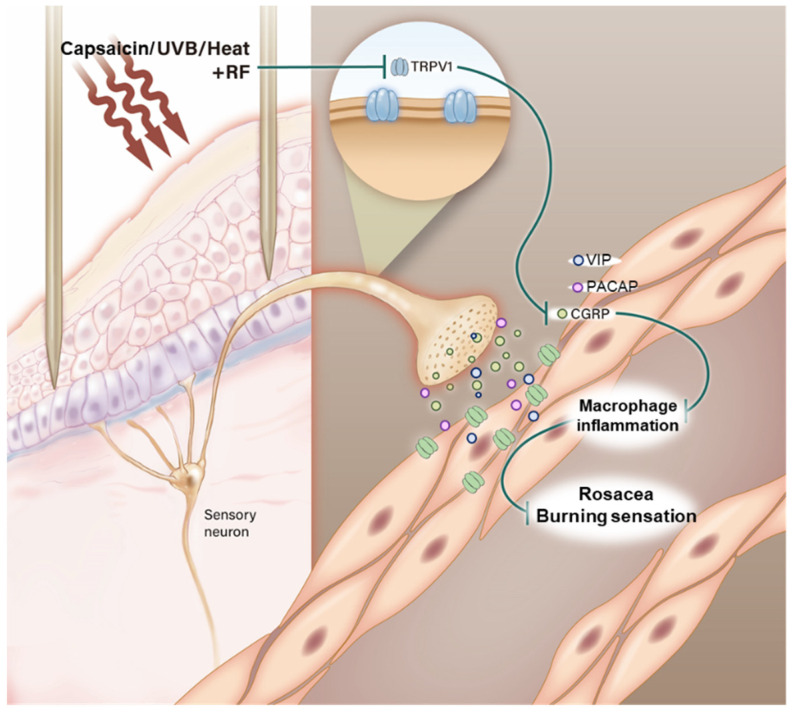
Illustration shows how RF irradiation in Capsaicin, UVB or heat-treated sensory neurons decreased neuropeptides including CGRP, PACAP and VIP and these molecules modulated CD163-positive macrophage and inflammation. Finally, facial temperature of Rosacea patients effectively decreased.

In general, the facial temperature of patients with rosacea is warmer, compared with that of normal skin, because of redness and flushing. Moreover, the most uncomfortable symptom in patients with rosacea is a burning sensation on the skin. In this study, the facial temperature and complaints of patients with rosacea decreased after every RF irradiation (Figure 5). Empirically, we found that clinical application of RF irradiation, similar to pulsed dye lasers or IPL, can improve rosacea lesions. This study provided experimental evidence that supported the use of RF for rosacea treatment (Figure 6).

## 4. Materials and Methods

### 4.1. Cell Culture

#### 4.1.1. Sensory Neuron Cells (F11)

Sensory neuronal cell lines (F11, ATCC, Manassas, VA, USA) were grown in Dulbecco′s Modified Eagle′s Medium (DMEM, Hyclone, Logan, UT, USA) with high glucose, 10% fetal bovine serum (FBS, Merk, Fort Kennerworth, NJ, USA), 5% calf serum, and 1% penicillin/streptomycin (P/S, Thermo scientific, Waltham, MA, USA) at 37 °C under 5% CO_2_.

#### 4.1.2. Macrophage Cells (Raw 264.7)

Macrophage cell lines (Raw 264.7, Korean Cell Line Bank) were cultured with high-glucose DMEM, 10% FBS, and 1% P/S (Thermo scientific) at 37 °C under 5% CO_2_.

### 4.2. Experimental Cell Models

#### 4.2.1. Heat Treatment Model

The F11 cells underwent heat treatment at 45 °C for 7 min before RF irradiation (2 MHz; 10 Watts; 50, 100, or 150 ms) and culture for 24 h. After 24 h, the F11 cells were harvested, and the heat-conditioned media were retrieved. The collected heat-conditioned media were treated with Raw 264.7 cells, incubated for 24 h, and harvested after 24 h.

#### 4.2.2. UVB Exposed Model 

The F11 cells were exposed to UVB for 15 min before RF irradiation (2 MHz; 10 Watts; 50, 100, or 150 ms) and culture for 24 h. After 24 h, the F11 cells were harvested, and UVB-conditioned media were retrieved. The collected UVB-conditioned media were treated with Raw 264.7 cells, incubated for 24 h, and harvested after 24 h.

### 4.3. UVB-Induced Rosacea Mice Model

For the UVB-induced rosacea mice model in this study, 7-week-old male HRM-2 mice (3 mice per group) that were bred in a controlled facility with 12-h light–dark cycles were used. This study was approved by the Lee Gil Ya Cancer and Diabetes Institute of Gachon University. All the experiments were executed in accordance with the guidelines issued by the Institutional Animal Care and Use Committee (AAALAC; approval number LCDI-2018-0094; approval date 21 August 2018).

The mice models were exposed for five minutes in a UVB chamber every 2 days for 10 days; after 4 days, the mice were exposed UVB for five minutes every day. After observing skin pigments on the mice, RF was irradiated at 2 MHz, 10 W for 100 ms. After treatment, all the mice were exposed to UVB for five minutes every other day until the 28th day. All the mice were sacrificed for the skin sample.

### 4.4. Paraffin Tissue Preparation for Staining

#### 4.4.1. Paraffin Tissue Preparation

To fix the skin tissues, the samples were stored in 4% paraformaldehyde (Biosesang, Korea) at 4 °C overnight for two days. The fixed skin tissues were washed for 10 min before being passed through the tissue process machine (Shandon, Thermo fisher, Waltham, MA, USA) for 14 h, following the machine innate protocol. After completing the machine tissue process, the tissue was embedded in a paraffin block and stored at room temperature. The skin blocks were cut into 10-μm slices using microtome (Leica, Wetzlar, Germany) and dried at 37 °C overnight in a tissue warmer.

#### 4.4.2. Immunohistochemistry

To deparaffinize, the slides were placed twice in 100% xylene for 5 min. Thereafter, the tissue slides were placed in 100%, 95%, 90%, 80%, and 70% alcohol for 5 min each, and were placed through running water for three minutes for rehydration. Then, the slides were placed in an antigen retrieval solution for five minutes in the microwave before being submerged in cold water for 10 min. Subsequently, the slides were incubated in normal animal serum at room temperature for 1 h; washed thrice for 5 min; and incubated with TRPV1 (abcam; ab6166, 1:100 dilution) and CD163 (Santa Cruz Biotechnology; sc-58965, 1:200 dilution) primary antibodies at 4 °C. The slides were washed three times with phosphate-buffered saline (PBS) for five minutes and then incubated with biotinylated secondary antibodies (1:200 dilution) for 2 h at room temperature. After washing, the slides were incubated with the ABC kit (Vector Laboratory Inc., Burlingame, CA, USA) for 30 min. The tissue slides were washed with PBS then incubated in 3,3′-diaminobenzidine (DAB; Sigma Aldrich, St. Louis, MO, USA) for 8 min. After developing, the slides were washed in running water for 10 min then placed in hematoxylin (DAKO, Santa Clara, CA, USA) solution for 23 s. For dehydration, the slides were placed in 70%, 80%, 90%, 95%, and 100% alcohol for 5 min each. After mounting the stained slides with DPX solution (Sigma Aldrich) and cover glass, photomicrographs were taken by an optical microscope (BX51, Olympus Optical Co., Tokyo, Japan) and analyzed by Image J (NIH, Stapleton, NY, USA) program.

### 4.5. RNA Extraction of Mice Skin and Cells

The RNA in the skin tissue and cells of each group was extracted using 0.5 mL of RNiso (Takara, Kyoto, Japan). The homogenates were mixed with 0.1 mL of chloroform then centrifuged at 12,000 g for 15 min at 4 °C. Supernatants were collected and transferred into new tubes, onto which 0.5 mL of isopropanol was added before centrifuged under the same conditions. RNA pellets were washed with 0.5 mL of cold 75% ethanol and dissolved in 30 µL of diethyl pyrocarbonate-treated water. Complementary DNA (cDNA) was synthesized with 1 μg of RNA using Prime Script 1st strand cDNA Synthesis Kit (Takara, Kyoto, Japan).

### 4.6. Quantitative Real-Time Polymerase Chain Reaction (qRT-PCR)

Synthesized cDNA was analyzed by qRT-PCR (CFX 384 Touch Real-Time PCR detection system; Bio-rad, Hercules, CA, USA). The reaction mixtures were prepared in 384-well plates and cDNA template, and SYBR Green (Takara, Kyoto, Japan) was mixed with primers (Table 1).

### 4.7. RF Device

The system (Potenza, Jeisys medical Inc., Seoul, Korea) used for the animal experiments and clinical treatment was a bipolar pulsed-type electrode array RF device that allowed selection of the number of pulses (1 MHz or 2 MHz and monopolar or bipolar). An impedance matching system was applied to automatically measure the compensation value, and proper output was irradiated through a 16-Ea (4 × 4) needle tip. Alternating current oscillations emitting 2-MHz pulsed-type bipolar RF were used in the animal experiment (type A) and clinical treatment (type B). Single pulse-type bipolar RF devices were used for type A and comprised on-time pulse duration of 100 ms at a power density of 10 W/pulse. Dual pulse-type bipolar RF devices were used for type B and comprised on-time pulse duration of 50 ms and off-time pulse duration of 30 ms at a power density of 5 W/pulse. The duty cycle was 130 ms. We conducted the investigation and treatment with 10 mm × 10 mm disposable tips that comprised 16 invasive microneedle electrodes, which had length of 13.6 mm, diameter of 250 mm, and a needle-to-needle distance of 1.3 mm. This tip type was approved by NAMSA (Northwood, OH, USA) through a biological compatibility test.

#### 4.7.1. RF Treatment for Animal Experiments

Single pulse-type bipolar RF devices were used for animal study and comprised on-time pulse duration of 100 ms at a power density of 10 W/pulse. 

#### 4.7.2. RF Treatment for Clinical Treatments and Measuring Facial Temperature 

Dual pulse-type bipolar RF devices were used for clinical treatments and comprised on-time pulse duration of 20 ms and off-time pulse duration of 10 ms at a power density of 2 W/pulse. Totally, RF was performed 5 times (total 100 ms, 10 W) of 20 ms each time with 2W power. Patients visited for a total of 5 months and performed the procedure at the 1st, 2nd, 3rd, and 4th rounds before the procedure, and at the 5th round, the temperature of the facial skin was measured without the RF treatment.

### 4.8. Statistical Analysis

To validate the significance of difference among animals, the D’Agostino and Pearson omnibus normality test was performed and then, the Kruskal–Wallis test was used for comparisons of 5 groups, followed by Mann–Whitney U as a post-hoc test. Human study was validated by paired t test. All the results are presented as median ± range and statistical significance was accepted for *p* < 0.05. The statistical analysis was executed by SPSS version 22 (IBM Corporation, Armonk, NY, USA) and each figure legend informs the *p* values. All experiments were conducted 3 times, and 3 repetitions were performed per experiment.

## Figures and Tables

**Table 1:** List of primers for quantitative real time polymerase chain reaction (qRT-PCR).

Gene Name	Primer Sequence	
*Actb*	Forward	5′-ACA AAG CTG TTC AGT GTC TCC A-3′
	Reverse	5′-CTC CGT TTC CAG AAT ACA CAC A-3′
*T* *rpv1*	Forward	5′-GGC TAG CTT AGA CTC GGA TGA A-3′
	Reverse	5′-GGAGTTAGGGTCTCTGTCTGGA-3′
*Camkii*	Forward	5′-CCT GGT GTT GCT AAC CCT CTA C-3′
	Reverse	5′-ATT CCT TCA CAC CAT CGT TCT T-3′
*Pkc*	Forward	5′-AGA CTG CAC CCT CGT AGA GAA G-3′
	Reverse	5′-AGT CTG AAA GGT GGA GTG AAG C-3′
*Pacap*	Forward	5′-CGC TGC AAG ACT TCT ATG ACT G-3′
	Reverse	5′-ATA GTA AAG GGC GTA AGC GTC A-3′
*Vip*	Forward	5′-ATA ACT ACA CCC GCC TCA GAA A-3′
	Reverse	5′-AAT CTC CCT CAC TCC TCT TTC C-3′
*Cd163*	Forward	5′-TGC CAT GTA GTT CAT TGT CTC C-3′
	Reverse	5′-GCT ATG CAG GGA ACT TCA CTC T-3′
*Il-1β*	Forward	5′-CTT TTC GTG AAT GAG CAG ACA G-3′
	Reverse	5′-TCA GCT TCA ATG AAA GAC CTC A-3′
*Tnf-α*	Forward	5′-TTC TGT CTA CTG AAC TTC GGG GTG ATC GGT CC-3′
	Reverse	5′-GTA TGA GAT AGC AAA TCG GCT GAC GGT GTG GG-3′
*inos*	Forward	5′-CAC AGC AAT ATA GGC TCA TCC A-3′
	Reverse	5′-AGC CTC ATG GTA AAC ACG TTC T-3′

## Data Availability

All data is contained within the article.

## Supplementary Materials

The following are available online at Figure S1. Inhibitory effects of RF on TRPV1 and PKC-dependent pathway in capsaicin-treated sensory neuronal cells; Figure S2. Inhibitory effects of RF on TRPV1-induced neuropeptide expression in capsaicin-treated sensory neuronal cells.

## Sample Availability

Samples of the compounds ore not available from the authors.
