# Supplementary material for: Radiofrequency Irradiation Modulates TRPV1-Related Burning Sensation in Rosacea
Source: Molecules. 2021 Mar 6;26(5):1424. doi: 10.3390/molecules26051424 (PMC7961329; doi:10.3390/molecules26051424)
Supplement: Supplementary file 1 [file molecules-26-01424-s001.pdf]

Article

Radiofrequency Irradiation Modulates TRPV1-Related Burning Sensation in Rosacea

Seyeon Oh [1,†], Myeongjoo Son [1,2,†], Joonhong Park [3], Donghwan Kang [4] and Kyunghee Byun [1,2,*]

[1] Functional Cellular Networks Laboratory, Lee Gil Ya Cancer and Diabetes Institute, Gachon University College of Medicine, Incheon, Republic of Korea; seyeon8965@gmail.com (S.O.); mjson@gachon.ac.kr (M.S.)
[2] Department of Anatomy & Cell Biology, Gachon University College of Medicine, Incheon, Republic of Korea
[3] Maymorning Dermatologic Clinic, Sungnam, Republic of Korea; 5maymorning@gmail.com
[4] Jeisys Medical Inc., Seoul, Republic of Korea; kang@jeisys.com
* Correspondence: khbyun1@gachon.ac.kr; Tel.: +82-32-899-6511
† These authors contributed equally to this work.

Capsaicin treatment model

The F11 cells were treated capsaicin 10μM for 10 minutes [S1] before RF irradiation (2 MHz; 10 Watts; 50, 100, or 150 ms) and culture for 24 hours. After 24 hours, the F11 cells were harvested.

Supplementary figure

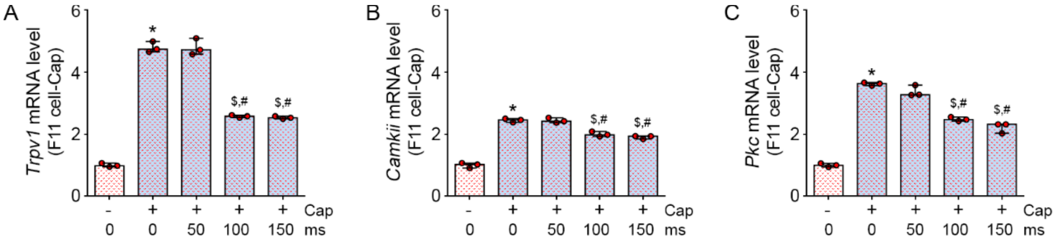

Figure S1. Inhibitory effects of RF on TRPV1 and PKC-dependent pathway in capsaicin-treated sensory neuronal cells. (A-C) Expression of the TRPV1 related PKC-dependent pathway molecule, *Camkii* and *Pkc* mRNA were confirmed by qRT-PCR in capsaicin treated F11 cell model and were normalized to that of *Actb* and expressed relative to the control group. *, $p < 0.05$ vs. Control; $, $P < 0.05$ vs. Capsaicin; #, $P < 0.05$ vs. RF 50ms. Cap, Capsaicin.

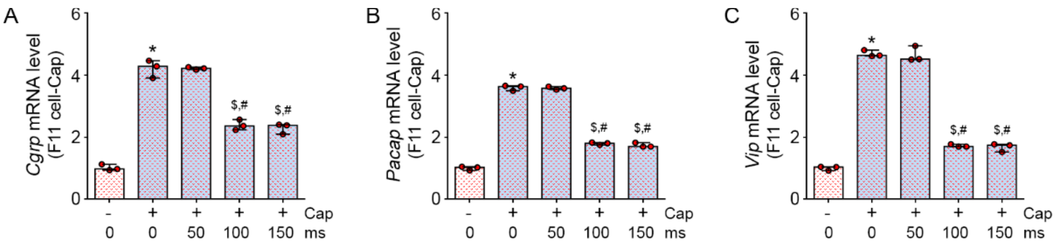

Figure S2. Inhibitory effects of RF on TRPV1-induced neuropeptide expression in capsaicin-treated sensory neuronal cells. (A–C) TRPV1-induced neuropeptide (*Cgrp*, *Pacap* and *Vip*) mRNA expression level was analyzed by qRT-PCR in capsaicin treated F11 cell model and were normalized to that of *Actb* and expressed relative to the control group. *, $p < 0.05$ vs. Control; $, $P < 0.05$ vs. Capsaicin; #, $P < 0.05$ vs. RF 50ms. Cap, Capsaicin.

Supplementary reference

S1 Erine F.; Scott K-G.; Trevor K.; Jeff M.; Aaron D. TRPV1 Agonist, Capsaicin, Induces Axon Outgrowth after Injury via Ca^{2+}/PKA Signaling. *eNeuro* **2018,** *5*, ENEURO.0095-18.
